# Histological analysis of arteriovenous anastomosis-like vessels established in the corpus luteum of cows during luteolysis

**DOI:** 10.1186/s13048-016-0277-0

**Published:** 2016-10-19

**Authors:** Junko Nio-Kobayashi, Kaya Miyazaki, Kazuhisa Hashiba, Kiyoshi Okuda, Toshihiko Iwanaga

**Affiliations:** 1 Laboratory of Histology and Cytology, Hokkaido University Graduate School of Medicine, Kita 15-Nishi 7, Kita-ku, Sapporo, 060-8638 Japan; 2 Laboratory of Reproductive Physiology, Graduate School of Environmental and Life Science, Okayama University, Tsushima Naka Kita-ku 1-1-1, Okayama, 700-8530 Japan; 3 Obihiro University of Agriculture and Veterinary Medicine, 2-11, Nishi, Inada-cho, Obihiro, 080-8555 Japan

**Keywords:** Luteolysis, Arteriovenous anastomosis, Myofibroblasts, PGF_2α_, TGFβ1

## Abstract

**Background:**

The mechanisms regulating the function and regression of the corpus luteum (CL) have not yet been elucidated in detail. The regressed CL of cows was previously reported to be filled with unusual vessels like arteriovenous anastomosis (AVA); however how these vessels are being established during luteolysis remains unknown.

**Methods:**

The bovine CL at different luteal stages and regressing bovine CL induced by prostaglandin F_2α_ (PGF) were histologically analyzed using light and electron microscopic levels. The changes in mRNA expression of genes encoding α-smooth muscle actin (SMA; *Acta2*) and transforming growth factor β1 (*Tgfb1*) in luteal tissues were analyzed by quantitative RT-PCR.

**Results:**

AVA-like vessels appeared in the regressed CL with a diameter less than 1.5 cm in which no functional luteal cells and macrophages were observed. Epithelioid cells in the AVA-like vessel wall were immunoreactive for SMA, and the lumen of the vessels were narrow. Immunoreaction for SMA was found in the tunica media of typical arteries and arterioles, and pericytes around capillary vessel. Cells with elongated cytoplasmic processes ―resident fibroblasts expressing vimentin― distributed in the CL parenchyma without any association with blood vessels are also immunoreactive for SMA, and accumulated around arteries and arterioles during the late-luteal stage. In the regressed CL, walls of arteries and arterioles consisted of more than two layers of epithelioid cells positive for both SMA and desmin, suggesting that they are myofibroblasts transformed from fibroblasts. The percentage of the area positive for SMA and the mRNA expression of *Acta2* were significantly increased in the regressed CL; however, they did not alter when a luteolytic dose of PGF was injected in vivo and collected within 24 h after the injection. On the other hand, *Tgfb1*, a known regulator for myofibroblast transformation, was significantly increased in PGF-induced regressing CL as well as in the CL during the late-luteal stage.

**Conclusions:**

SMA-positive myofibroblasts accumulates around the arteries and arterioles to form AVA-like vessels during luteolysis in cows. PGF indirectly regulates myofibroblast transformation through enhancing the expression of TGFβ1. These peculiar AVA-like vessels may be involved in the regulation of blood flow in the bovine CL during luteolysis.

## Background

The corpus luteum (CL) is formed from an ovulated dominant follicle in the ovary, and secretes progesterone, which is essential for the establishment and maintenance of pregnancy. In mammals, the CL undergoes luteolysis at the end of luteal phase [1]. There are two phases in luteolysis: functional and structural luteolysis. Functional luteolysis is characterized by a decrease in serum progesterone concentrations and is followed by structural luteolysis, in which the volume of the CL decreases due to apoptotic death of luteal cells. It is generally accepted that spontaneous luteolysis in cows is initiated by uterus-derived prostaglandin F_2α_ (PGF) if pregnancy does not occur [2]. In cows, serum concentration of progesterone markedly decreases approximately 19 days after ovulation and this is accompanied by an increase in plasma PGF metabolites, which represents the production of PGF in the uterus [3]. An intramuscular injection of PGF into cows during the mid-luteal stage ―but not the early-luteal stage― has been shown to induce luteolysis, resulting in a rapid decrease in serum progesterone levels eight hours after the injection [3, 4]. Although PGF is a major luteolysin in cows, the molecular mechanisms by which PGF regulates functional and structural luteolysis currently remain unclear; however, its effects are known to be mediated by various intra-ovarian factors, such as endothelin 1, nitric oxide, angiotensin II, and interferon γ [5–11].

In cows, ovarian blood flow decreases during luteal regression [4, 12] and the resulting hypoxic condition affects functional and structural luteolysis [13] although a temporal increase in blood flow has been reported in spontaneous and PGF-induced luteolysis [3]. Previous studies noted morphological changes in the vasculature during luteolysis in ewes [14] and cows [15]. Sawyer et al. [14] found that vascular occlusion occurred following the sloughing of endothelial cells into the lumina of small blood vessels during luteolysis, suggesting that vascular occlusion causes a decreased blood supply, resulting in hypoxic conditions in the CL. Hojo et al. [15] demonstrated that capillaries disappeared within the CL at the late and regressed luteal stages, whereas larger blood vessels that stained for α-smooth muscle actin (SMA) increased in number. Changes in the vascular structure have been suggested to contribute to a decrease in blood flow and luteolytic process in the CL.

Although remodeling of the vasculature in the CL appears to be essential in the regulation of luteal function during luteolysis, the mechanisms responsible for controlling changes in vascular structures in the bovine CL during luteolysis have yet to be clarified. Höflinger [16] noted the existence of arteriovenous anastomosis (AVA)-like vessels in the regressed CL of cows. AVA is characterized by precapillary connections between small arteries and veins. AVA called Hoyer-Grosser’s organs are distributed in the subcutaneous tissue of the tips of the fingers and toes of humans, and have also been reported in the ears of some animals including rabbits. The corpora cavernosa penis of various animals possesses a similar structure in the helicine arteries [17–19]. AVA is a vascular shunt and is involved in the regulation of blood flow in the skin or corpora cavernosa penis. Without stimulations by vasodilative molecules such as nitric oxide secreted from peripheral nerves, AVA contracts and blood flow is remarkably obstructed. AVA shows a characteristic morphology that is markedly different from those of normal arteries and veins: unique round epithelioid cells form the walls of AVA. Epithelioid cells are contractive cells containing SMA in the cytoplasm, and react to both vasoconstrictive and vasodilative signals such as endothelin and nitric oxide [20].

Because the blood flow is known to decrease during luteolysis and the appearance of AVA-like vessels is likely to contribute to a decrease in blood flow in the CL of cows during luteolysis; however, the role of AVA-like vessels in regulating the function of CL and how this structure is constructed in the CL remain unclear. The objective of this study is to clarify the contribution of AVA-like vessels to the regulation of luteal function in cows, we herein investigated how they are formed in the bovine CL during luteolysis using histological techniques.

## Methods

### Tissue collection

Ovaries with the CL of Holstein cows were obtained at a local abattoir, kept in normal saline at 25 − 30 °C, and delivered to the laboratory within 6 h of being collected. Luteal stages were classified as early-luteal (days 2–3 after ovulation), mid-luteal (days 8–12), late-luteal (days 15–17), and regressed (over day 19) by macroscopic observations of the CL according to a previous study [21]. The stages of the bovine CL were classified into the early-luteal (*n* = 7), mid-luteal (*n* = 13), late-luteal (*n* = 14), and the regressed stages (*n* = 31). Collection of the CL tissues was approved by the local ethical committee of Hokkaido University (Approval no. 13–0052).

The animal procedures used to collect PGF-induced regressing CL tissues were approved by the local Institutional Animal Care and Use Committee of the Polish Academy of Science in Olszyn, Poland (Agreement No. 5/2007, 6/2007 and 88/2007). Healthy, normally cycling Polish Holstein black and white cows were used for the collection of PGF-induced regressing CLs. Estrus was synchronized in cows by two injections of an analogue PGF (25 mg; Dinoprost, Dinolytic; Pharmacia and Upjohn, Puurs, Belgium) with an 11-day interval according to the manufacturer’s instructions. Ovaries were collected 0, 2, 12, and 24 h after the injection of a luteolytic dose of the PGF analogue (25 mg) on post-ovulation day 10 using a Hauptner’s effeninator (Hauptner & Herberholz, Solingen, Germany). Total 25 PGF-treated CL and control mid-luteal CL were used in this study, and detailed information of the tissues is summarized in Table 1.

**Table 1:** Information on the bovine corpus luteum used for the present study

A: Normal cycling CL					
	Luteal stages				
		EL	ML	LL	Reg
Number of samples	For the histological analysis	7	13	14	31
	For the qPCR analysis	4	4	5	7
Days after ovulation		2–3	8–12	15–17	>19
Color		Flesh, Bloody	Orange	Yellow	Ocher, Brown, White
Size (cm)		1.9 ± 0.19	2.5 ± 0.07	2.1 ± 0.14	0.9 ± 0.06
B: PGF-induced regressing CL					
		Hours after the PGF injection			
		0	2	12	24
Number of samples	For the histological analysis	4	10	6	3
	For the qPCR analysis	5	6	6	4

*EL* early-luteal, *ML* mid-luteal, *LL* late-luteal, *Reg* regressed

CL tissues dissected out from the ovaries were halved, and either immersed in fixatives as described below for the histological analysis or frozen in liquid nitrogen for RNA purification. Information on the CL used for this study was summarized in Table 1.

### Histological analysis

CL tissues were dissected from the ovaries and immersed in 4 % paraformaldehyde for 24 h at 4 °C. Tissues were dehydrated through graded series of ethanol and immersed into xylene, then embedded in paraffin according to the standard methodology, and 5-μm-thick sections were obtained. Sections were de-waxed, subjected to Hematoxylin and Eosin staining, and observed under a light microscope (BX51; Olympus corporation, Tokyo, Japan). The middle CL parenchyma tissues were dissected into small pieces and fixed with 2.5 % glutaraldehyde for ultrastructural observations under a transmission electron microscope (TEM, H-7100; Hitachi, Tokyo, Japan). Tissue pieces were post-fixed with 1 % OsO4 for 1.5 h, dehydrated through a graded series of ethanol, and embedded into Epon resin (Quetol 812). Ultra-thin sections were obtained, stained with uranyl acetate lead citrate, and observed under a TEM.

### Immunohistochemistry

All procedures were performed at room temperature. After deparaffinization, endogenous peroxidase was blocked by 3 % H_2_O_2_ in distilled water for 20 min. Sections were then incubated with Avidin/Biotin blocking solution (Vector Laboratories Inc., Burlingame, CA, USA) for 15 min each. After washing with phosphate-buffered saline (PBS) twice, sections were pre-incubated for non-immune blocking with Block Ace (DS Pharma Biomedical Co., Ltd., Osaka, Japan) for 60 min. Sections were then incubated with a mouse anti-SMA antibody (1:1,000; clone 1A4, #A2574; Sigma-Aldrich Corporation, St. Louis, MO, USA) in PBS overnight. The sections were incubated with biotinylated anti-mouse IgG (1:500; Vector Laboratories Inc.) for 1 h, followed by an incubation using a Vectastain ABC Elite kit (Vector Laboratories Inc.) for 1 h. The reaction site of the primary antibody was visualized using an ImmPACT™ DAB Peroxidase Substrate Kit (Vector Laboratories Inc.) for 3 min. Sections were counterstained with hematoxylin and observed under a light microscope. The total CL area in the section and the SMA-immunoreactive area in the CL were measured using Image J (http://imagej.nih.gov/ij/), and the percentage of SMA-positive area per the total CL area was calculated. Control sections were incubated with PBS instead of the primary antibody and the disappearance of the signals was confirmed.

For luteal steroidogenic cell, macrophage, and proliferating cell staining, antigen retrieval was performed for 1 min in boiling 0.01 M citrate buffer (pH 6.0) using a pressure cooker. Endogenous peroxidase and Avidin/Biotin blocking were then performed as described above. After a pre-incubation with Block Ace for 1 h, the sections were incubated with either goat anti-3β-hydroxysteroid dehydrogenase (3β-HSD) (1:2,000; #sc-30820; Santa Cruz Biotechnology, Inc., Dallas, TX, USA), mouse anti-MAC387 (1:1,000; #ab22506; Abcam Japan Ltd., Tokyo, Japan), which recognizes the L1 or Calprotectin molecule, an intracytoplasmic antigen expressed by tissue macrophages, or rabbit anti-Ki-67 antibody (1:200; ThermoFisher Scientific Inc., Waltham, MA, USA) overnight. Sections incubated with the 3β-HSD antibody were then incubated with a peroxidase-labeled anti-goat IgG (1:200; Vector Laboratories Inc.) for 1 h. On the other hand, MAC387- and Ki-67-stained sections were incubated with biotinylated anti-mouse or rabbit IgG for 1 h and Vecterstain ABC Elite kit as described above. Reaction sites for all three antibodies were visualized using an ImmPACT™ DAB Peroxidase Substrate Kit (Vector Laboratories Inc.) for 3 min and observed under a light microscope as described above. The total CL area in the section and the number of 3β-HSD- or MAC387- immunoreactive cells in the CL was measured using Image J, and the positive cell number in 1 mm^2^ was calculated.

For double immunostaining for SMA and Ki-67, the deparafinized sections were rinsed in distilled water and antigen retrieval was performed by boiling the sections in 0.01 M citrate buffer (pH 6.0) for 1 min using a pressure cocker. The sections were incubated with Block Ace for 1 h and rabbit anti-Ki-67 antibody overnight as described above. After washing with PBS three times, the sections were incubated with Alexa Fluor 488-conjugated anti-rabbit IgG (1:200; Life Technologies Japan, Tokyo, Japan) for 2 h. The sections were again incubated with Block Ace for 1 h and then incubated with mouse anti-SMA antibody as described above. The binding site for SMA antibody was visualized using AlexaFluor 594-labeled anti-mouse IgG (1:200; Life Technologies Japan) and observed under a confocal laser microscope (FV300; Olympus, Tokyo, Japan).

For triple staining, sections were incubated with a mouse anti-vimentin antibody (1:500; Dako Japan ltd., Tokyo, Japan) overnight, and then with biotinylated anti-mouse IgG for 1 h as described above. The antibody-binding site for the 1^st^ primary antibody against vimentin visualized using the TSA™ Fluorescein System (PerkinElmer Inc., Waltham, MA, USA). The 1^st^ primary antibody was washed out in boiling 0.01 M citrate buffer (pH 6.0) using a microwave for 2.5 min, and sections were then blocked with Block Ace for 1 h. They were incubated with the 2^nd^ primary antibody, a rabbit anti-desmin antibody (1:500; ThermoFisher Scientific Inc.) overnight and with a Cy5-labeled anti-rabbit antibody (1:200; Jackson ImmunoResearch Laboratories, Inc., West Grove, PA, USA) for 2 h. Sections were again blocked with Block Ace for 60 min and then incubated with a 3^rd^ primary antibody, the mouse anti-SMA antibody (1:500). The binding site for the 3^rd^ primary antibody was visualized by AlexaFluor 594-labeled anti-mouse IgG (1:200; Life Technologies Japan). Sections were observed under a confocal laser microscope as described above. Lasers used in this study were Ar (488 nm), HeNe (G) (543 nm), and HeNe (R) (633 nm).

### Quantitative PCR (qPCR)

Total RNA from bovine CL tissues during the early-luteal (*n* = 4), mid-luteal (*n* = 4), late-luteal (*n* = 5), and regressed stages (*n* = 7) was purified using Trizol® (Thermo Fisher Scientific) according to the manufacturer’s instructions. Two hundred nanograms of total RNA were used to prepare cDNA using a QuantiTect Reverse Transcription kit (Qiagen Japan Co. Ltd., Tokyo, Japan). QPCR was performed using a KAPA SYBR First qPCR kit (Nippon Genetics Co., Ltd., Tokyo Japan) according to the manufacturer’s instructions. The sequences of the primer sets used for this study are listed in Table 2. Primers were pre-validated by standard PCR and by generating standard curves using qPCR. The qPCR cycling program consisted of a denaturing step (95 °C for 3 min), annealing and extension step (95 °C for 3 s and 60 °C for 20 s repeated for 40 cycles), and a dissociation step (ramp from 60 to 95 °C) using Corbett Rotor-gene 6000 (Qiagen Japan Co, Ltd.). The relative expression levels of each target to the housekeeping gene (*glyceraldehyde 3-phosphate dehydrogenase*: *Gapdh*) were quantified using the ^Δ^Ct method.

**Table 2:** Primers used for the present study

Gene name	Accession no.	Forward	Reverse	Product size
*Gapdh*	BC102589	caccctcaagattgtcagca	ggtcataagtccctccacga	103
*Acta2*	NM_001034502	gccgagatctcaccgactac	gtgatcacctgcccatcag	202
*Tgfb1*	NM_001166068	ccttcctgctcctcatgg	ggttgtgctggttgtacagg	276

### Statistical analysis

After testing for normality, all statistical analyses were performed using one-way ANOVA with Tukey’s multiple comparisons test using GraphPad Prism 6 software (GraphPad Software Inc., San Diego, CA, USA), and *P* < 0.05 was regarded as significant. Values in the graphs represent the mean ± SEM.

## Results

### Characteristic AVA-like vessels appear in the regressed CL of cows

**Fig. 1:**
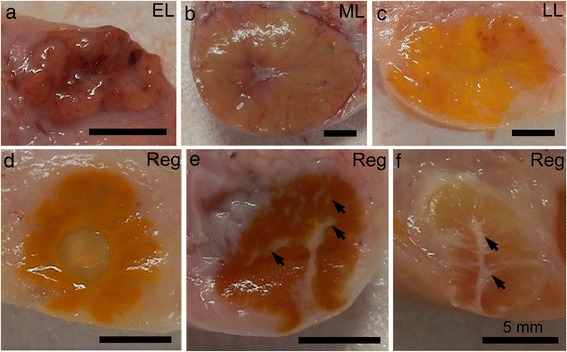
Macroanatomical images of the bovine CL. The CL was cut through an equatorial plane. The color of the CL during the early-luteal (EL) stage is bloody or flesh and size was less than 2.0 cm (**a**). The CL during the mid-luteal (ML) and late-luteal (LL) stages increased in size to more than 2.0 cm in diameter (**b**, **c**). The color of the CL during the ML stage is flesh or orange (**b**), while that during the LL stage turns to yellow (**c**). Regressed CLs are small with a diameter of less than 1.5 cm and show various colors such as ocher (**d**), brown (**e**), or white (**f**). Arrows in E and F show connective tissue in the CL parenchyma

Figure 1 shows macroscopic images of the equatorial plane of the bovine CL at each stage. The CL at the early-luteal stage displayed a flesh or bloody color and was very soft (Fig. 1a). The CL during the mid-luteal and late-luteal stages was large with a diameter of more than 2.0 cm (Fig. 1b, c). The color of the CL at the mid-luteal stage was flesh or orange while that at the late-luteal stage was yellow (Fig. 1b, c). The regressed CL with a diameter less than 1.5 cm showed various colors, such as ocher, brown, or white (Fig. 1d–f). The color of the regressed CL occasionally resembled that of the CL at the early-luteal phase, while the regressed CL was harder and possessed septa of connective tissue in the CL parenchyma (arrows in Fig. 1e, f).

**Fig. 2:**
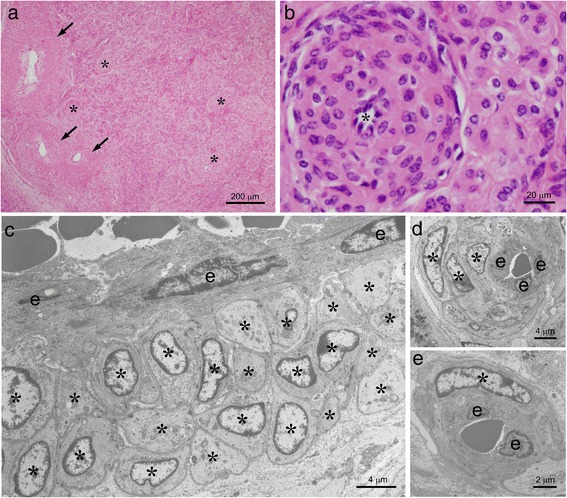
Histological features of AVA-like vessels in the regressed CL of cows. The regressed CL of cows is filled with AVA-like vessels. Large vessels with an external diameter of more than 200 μm are found in the periphery of the CL (*arrows* in **a**) and vessels of various sizes are distributed throughout the CL parenchyma (**a**). Vessels with a diameter of 100–200 μm resemble typical AVA possessing several layer of epithelioid cells in the walls and narrow lumens (asterisks in **a**, **b**). Ultrastructural observations under a TEM show how the epithelioid shape of cells in wall of an AVA-like vessel, each possessing a basement membrane similar to smooth muscle cells (asterisks in **c**). The lumen of small-sized vessels is narrow, showing its contractive condition (**d**, **e**). e: endothelial cells

Under a light microscope, the parenchyma of the regressed CL was filled with characteristic vessels possessing thick walls (Fig. 2a). Large arteries with an external diameter of more than 200 μm possessed a thick tunica media and were located at the periphery of the CL (arrows in Fig. 2a). The CL parenchyma contained numerous and condensed arteries of various sizes (Fig. 2a, b). Vessels with a diameter of 100–200 μm, possibly modified small arteries, possessed more than four layers of SMA-positive epithelioid cells in their walls and a narrow lumen, possessing the features of typical AVA (asterisks in Fig. 2a, b). Observations of AVA-like vessels under an electron microscope clearly showed the characteristic structure of the vessels with a similar morphology to that of AVA, particularly in the small arteries (Fig. 2c). The walls of AVA-like vessels in the regressed CL consisted of round epithelioid cells, each of which possessed a basement membrane similar to smooth muscle cells (asterisks in Fig. 2c). The lumens of the meandering small-sized vessels were narrow, suggesting their contractive condition (Fig. 2d, e).

### SMA-positive cells increase in number during luteolysis

**Fig. 3:**
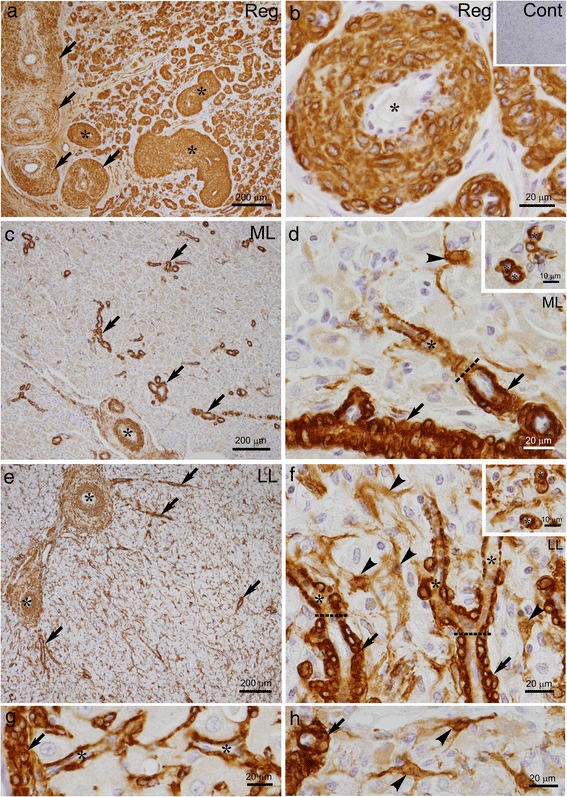
Representative staining of SMA in the CL during luteal stages. The walls of all vessels in the regressed (Reg) CL exhibited strong immunoreactivity for SMA (**a**, **b**). Tunica media of the large vessels at the periphery of the CL contains SMA immunoreactivity (*arrows* in **a**). Cells in the wall of typical AVA-like vessels with a diameter of 100–200 μm are immunolabeled with SMA (asterisks in **a**, **b**). In the CL during the mid-luteal (ML) stages, the immunoreactivity for SMA is largely restricted to the tunica media of arteries and arterioles (asterisks in **c** and arrows in **c**, **d**). Pericytes surrounding capillary vessels also immunoreactive for SMA (asterisk in **d**). Weak immunoreactivity for SMA is found in fibroblast-like cells lying between steroidogenic luteal cells but not being associated with blood vessels (arrowheads in **d**). During the late-luteal (LL) stage, abundant SMA-positive fibroblast-like cells are observed in the CL parenchyma and accumulate around the arteries (asterisks in **e**). Smooth muscle cells in the arteries (asterisks in **e**) and arterioles (arrows in **e**, **f**) as well as pericytes (asterisks in **f**) are immunolabeled with SMA. Capillary vessel network is more visible in the CL at the LL stage (asterisks in **g**). Fibroblastic cells with elongated cytoplasmic processes also contain SMA immunoreaction (arrowheads in **f**, **h**). Inserts in (**d** and **f**) show traverse images of capillary vessels. *Dotted lines* in (**d** and **f**) display changing points from arterioles to capillary vessels. Cont: negative control image

When the regressed bovine CL sections were immunostained for α-smooth muscle actin (SMA), the wall of the vessels in the CL parenchyma were all immunolabeled. Thick tunica media of the large vessels at the periphery of the CL was immunoreactive for SMA, whereas the enlarged tunica intima of the larger arteries was not (arrow in Fig. 3a). The wall of AVA-like modified arteries was strongly immunoreactive for SMA (asterisks in Fig. 3a, b). Small-sized vessels, possibly derived from arterioles and capillaries, occupied the CL parenchyma and displayed a spiral running pattern and intense immunoreactivity for SMA in their walls (Fig. 3a, b).

**Fig. 4:**
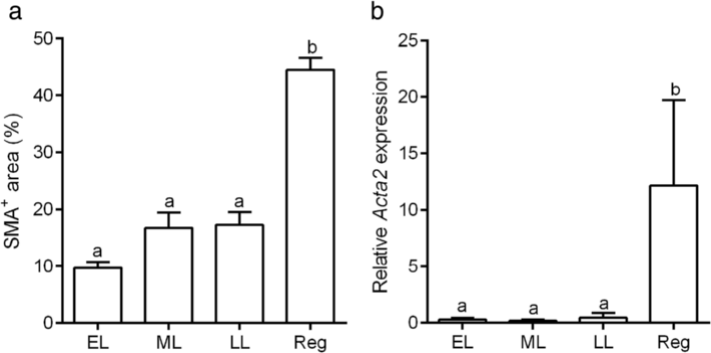
Change in SMA-positive area and *Acta2* mRNA expression in the CL during luteal stages. The percentage of the area immunoreactive for SMA (**a**) and the mRNA expression of *Acta2* significantly increase in the regressed CL (**b**). Different letters show significant differences

Because the wall of AVA-like vessels was immunostained with SMA, we next investigated how AVA-like vessels formed during luteal regression by SMA immunohistochemistry. Tissue sections of the bovine CL throughout the luteal phases were immunostained for SMA and observed with special reference to changes in the vascular structure. During the early- and mid-luteal stages, SMA-immunoreactive arteries and arterioles of various sizes were dispersed in the CL; typical arteries were located in the connective tissues surrounding the CL and septa within the CL (asterisks in Fig. 3c), and arterioles were observed throughout the CL parenchyma (arrows in Fig. 3c, d). Immunoreactivity for SMA was largely restricted to the tunica media of the arteries (asterisks in Fig. 3c) and arterioles (arrows in Fig. 3c, d) as well as cytoplasm of pericytes surrounding capillary vessels (asterisks in Fig. 3d). Other cells extending thin-cytoplasmic processes between luteal steroidogenic cells but not being associated with blood vessels occasionally showed weak immunoreactivity for SMA (arrowhead in Fig. 3d). During the late-luteal phase, the intensity of the SMA immunoreaction increased in these stellate cells (Fig. 3e, f, h). Furthermore, abundant SMA-positive cells appeared to accumulate around the arteries and arterioles (asterisks in Fig. 3e, f). Pericytes were strongly immunoreactive for SMA and the network of the capillary vessels were visible in the CL at the late-luteal stage (asterisks in Fig. 3f, g). The percentage of the SMA-positive area (*P* < 0.0001, Fig. 4a) was significantly increased in the regressed CL in accordance with the elevated mRNA expression of *Acta2* (*P* < 0.01, Fig. 4b).

**Fig. 5:**
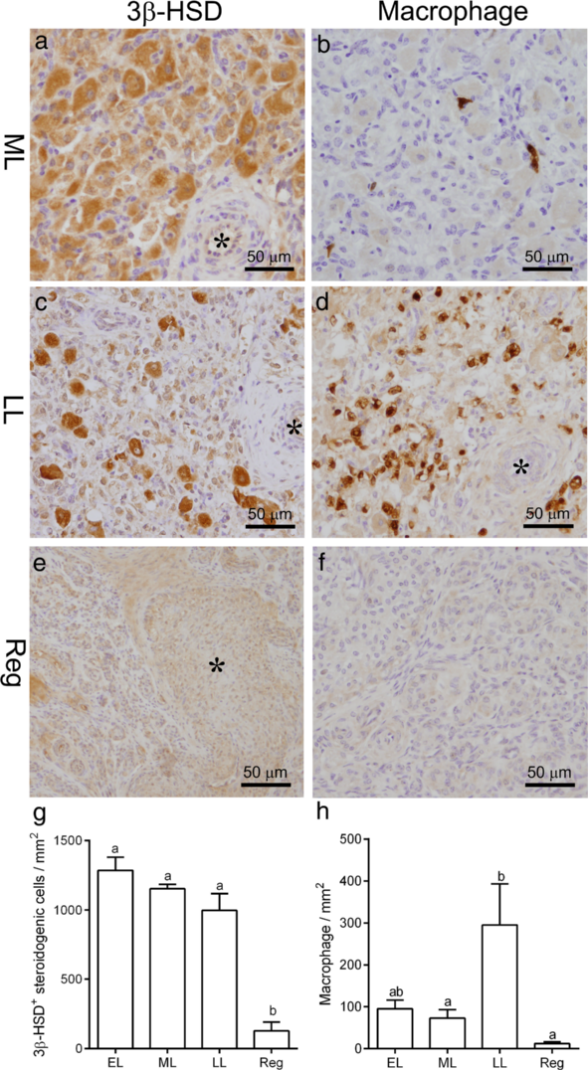
Change in numbers of 3β-HSD-positive steroidogenic cells and macrophage in the CL during luteal stages. Abundant luteal steroidogenic cells immunoreactive for 3β-HSD while a few number of MAC387-positive macrophages are found in the CL at the mid-luteal (ML) stage (**a**, **b**). In the CL at the late-luteal (LL) stage, 3β-HSD-positive cells scatters but abundant MAC387-positive cells are distributed (**c**, **d**). In the regressed (Reg) CL, both 3β-HSD-positive cells and MAC387-positive cells are negligible (**e**, **f**). The number of intact 3β-HSD-positive luteal steroidogenic cells significantly decreases in the regressed CL (**g**) while MAC387-positive macrophages increase in the CL at the LL stage (**h**)

In order to elucidate the relationship between the appearance of AVA-like vessels and luteal function, we immunostained the sections with markers for a key enzyme in progesterone production (3β-HSD) or macrophages (MAC387), which increase in number during luteolysis [22]. Luteal steroidogenic cells during the mid-luteal stage expressed 3β-HSD in the cytoplasm (Fig. 5a), while MAC387-positive macrophages were sparse (Fig. 5b). During the late-luteal phase, the number of MAC387-positive macrophages increased, whereas intact steroidogenic cells with the 3β-HSD immunoreaction was decreased (Fig. 5c, d). On the other hand, the number of 3β-HSD-positive intact luteal steroidogenic cells and MAC387-positive macrophages were both negligible in the regressed CL filled with SMA-positive AVA-like vessels (Fig. 5e, f). The number of 3β-HSD-positive steroidogenic cells was significantly low in the regressed CL (*P* ≤ 0.0001; Fig. 5g), whereas MAC387-positive macrophages increased in number in the CL at the late-luteal stage (*P* ≤ 0.05; Fig. 5h). These results indicate that αSMA-positive AVA-like vessels appear after the structural luteolysis.

### SMA-positive cells are myofibroblasts originating intraluteal fibroblast

**Fig. 6:**
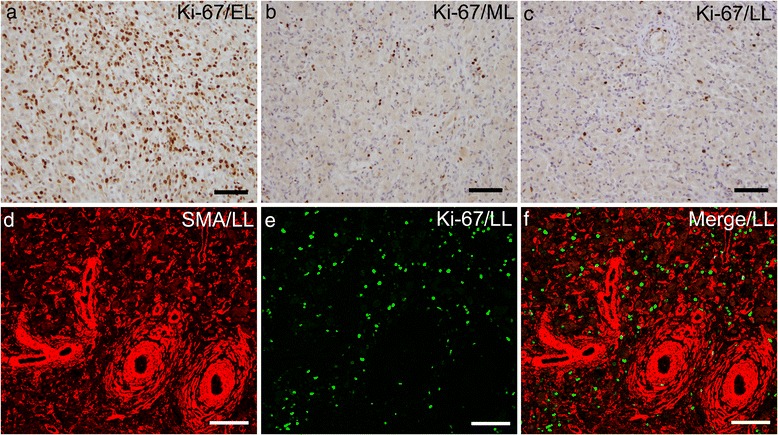
Dual immunostaining for SMA and a proliferation marker, Ki-67. Ki-67-positive proliferating cells are abundant in the CL at the early-luteal (EL) stage (**a**) but decreases during the mid-luteal (ML) and late-luteal (LL) stages (**b**, **c**). Most of SMA-positive cells (*red*) are not immunoreactive for Ki-67 (*green*) (**d**−**f**). Bars = 100 μm

**Fig. 7:**
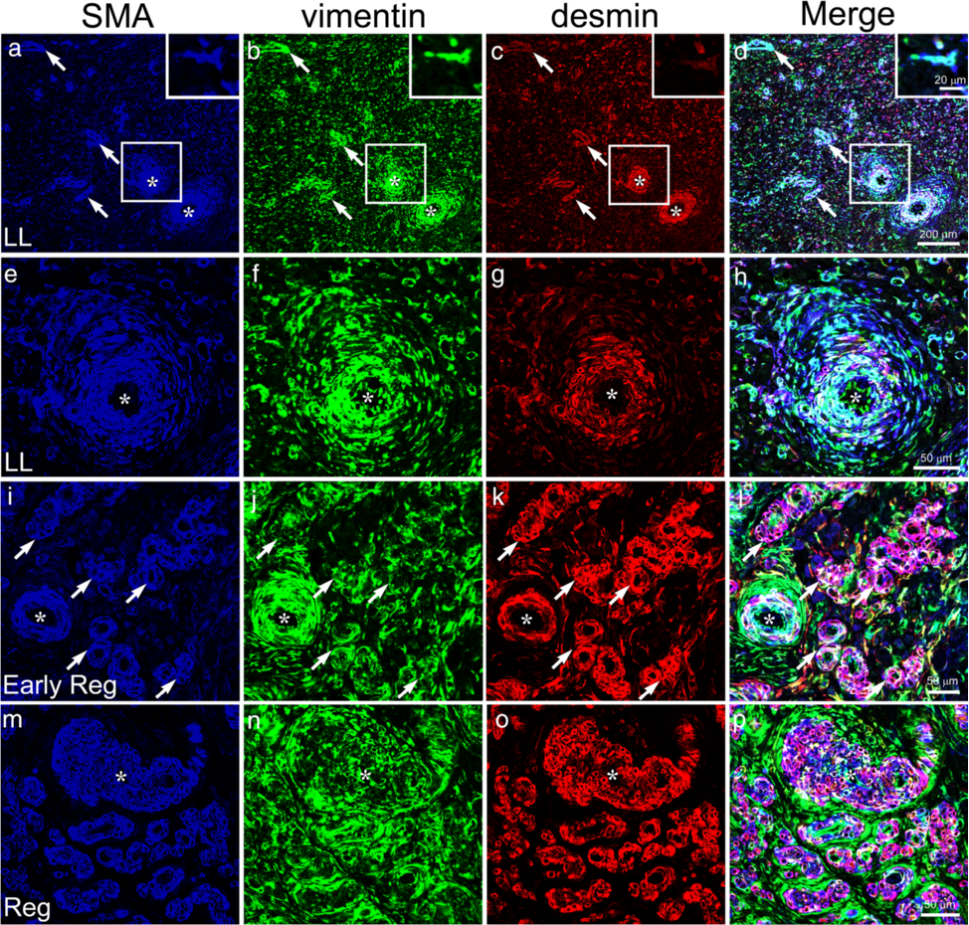
Triple immunohistochemical images for SMA, vimentin, and desmin in the bovine CL. In the CL during the late-luteal (LL) stage, the tunica media of arteries and arterioles of various sizes are immunoreactive for all three markers (asterisks and arrows in **a**–**d**). A specific number of SMA-positive cells distributed in the CL parenchyma contain vimentin (inserts in **a**–**d**), suggesting that they are resident fibroblasts. The tunica media of the artery contains desmin, a smooth muscle-specific intermediate filament, while immunoreactivities for SMA and vimentin are localized in the tunica media and cells loosely surrounding it (asterisks in **e**–**h**). In the early regressing CL, the walls of the arteries (asterisks in **i**–** l**) and crooked arterioles (arrows in **i**–**l**) abundantly contain both SMA and desmin, whereas the vimentin immunoreaction is strong outside the vessels (**i**–**l**). In the fully regressed CL, the thick walls of arteries (asterisks in **m**–**p**) and crooked vessels consist of SMA and desmin double-positive epithelioid cells. **e**–**h** is a closer view of the areas enclosed by squares in (**a**–**d**)

In order to investigate whether an increase in the number of SMA-positive stellate cells during luteolysis is due to cell proliferation or differentiation, sections of the bovine CL were immunostained with SMA and the proliferation marker, Ki-67. Ki-67-positive proliferative cells were abundant in the CL at the early-luteal stage (Fig. 6a) but decreased in number at the mid-luteal and late-luteal stages (Fig. 6b, c). Ki-67-positive cells scattered in the CL parenchyma and were not associated with SMA-immunoreactivity at the late-luteal stage (Fig. 6d–f). Most of Ki-67-positive proliferating cells seem to be luteal steroidogenic cells, endothelial cells, and immune cells as reported previously [23–26]. Thus, we concluded that resident cells ―possibly fibroblasts― express SMA during luteal regression. Triple staining for SMA, vimentin, and desmin at the late-luteal and regressing stages showed that certain numbers of SMA-positive cells distributed throughout the CL parenchyma were immunoreactive for vimentin, suggesting that they were resident fibroblasts (Fig. 7a-d). On the other hand, the small arteries in the intraluteal connective tissues and arterioles in the CL parenchyma were also immunolabeled with all three markers (asterisks and arrows in Fig. 7a-d). Immunoreactivity for desmin was restricted to the tunica media, whereas that for SMA and vimentin was detected in the desmin-positive tunica media and in cells loosely surrounding the arteries (asterisks in 7E–H). During the early regressing stage, in which luteal steroidogenic cells remained between the crooked vessels, SMA and desmin co-localized in the walls of the arteries and arterioles consisting of epithelioid cells (asterisks and arrows in Fig. 7i–l), while the vimentin immunoreaction was found in the walls of arteries, but was slightly more abundant outside vessels (Fig. 7j). In the fully regressed CL, the arterial wall became thicker and strongly expressed SMA and desmin, whereas immunoreactivity for vimentin was restricted more to the outside of these vessels (Fig. 7m–p). These results suggest that a certain population of vimentin-positive fibroblasts contain SMA, and transform into myofibroblasts that strongly express desmin during luteal regression, and accumulate around arteries to form AVA-like vessels.

### PGF-induced TGFβ1 may contribute to myofibroblast transformation

**Fig. 8:**
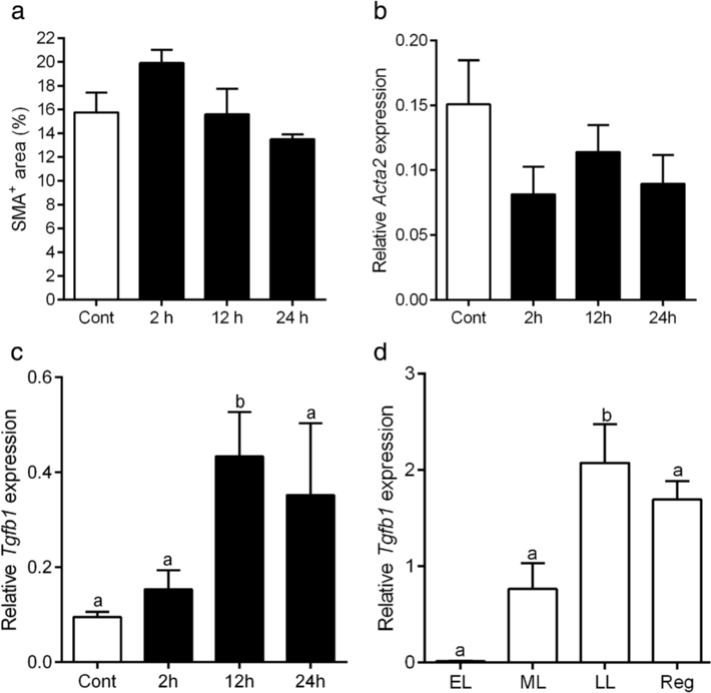
Effect of PGF on the expression of SMA and TGFβ1. The percentage of the SMA-positive area remains unchanged in the CL 2–24 h after the injection of a luteolytic dose of PGF (**a**). The mRNA expression of *Acta2* does not change by the PGF injection (**b**). On the other hand, the mRNA expression of *Tgfb1* ―a known regulator of myofibroblast transformation― is significantly increased in the CL 12 h after the PGF injection (**c**) as well as in the CL during the late-luteal (LL) stage and regressed stage (**d**). Cont: the CL not treated with PGF. ML: mid-luteal, Reg: regressed CL. *P < 0.05, **P < 0.01

Since PGF is a major luteolysin in cows, it may play a role in the transformation of fibroblasts to myofibroblasts during luteolysis and in the construction of AVA-like vessels. Therefore, we examined change in the number of SMA-positive cells and expression of *Acta2* mRNA in the CL collected 2–24 h after an injection of PGF. The staining pattern of SMA was similar to that in the CL during the mid-luteal stage, and the area positive for SMA in the CL and mRNA expression of *Acta2* were not affected by the injection of PGF (Fig. 8a, b). On the other hand, PGF significantly enhanced the expression of *transforming growth factor β1* (*Tgfb1*), which is known to be essential for the transformation of myofibroblasts [27] (Fig. 8c). The expression of *Tgfb1* was significantly increased during the late-luteal phase (*P* ≤ 0.01), and remained at a high level in the regressed stage (Fig. 8d), suggesting its contribution to luteolysis.

## Discussion

This study revealed the detailed morphology of AVA-like vessels appearing in the regressed bovine CL and how they are constructed during luteolysis. The regressed CL of cows contained abundant atypical arteries with thick walls of epithelioid cells that were strongly immunoreactive for SMA. These SMA-positive cells forming the walls of AVA-like vessels are myofibroblasts transformed from resident fibroblasts during luteolysis: SMA-expressing fibroblastic cells with elongated cytoplasmic processes increased in number during the late-luteal stage and accumulated around the arteries to form AVA-like vessels. Luteolytic PGF is unlikely to be a direct cause of this phenomenon, but indirectly regulate the transformation of fibroblasts into myofibroblasts by enhancing the expression of TGFβ1, a potent regulator for myofibroblast transformation.

It is well known that the CL development is associated with angiogenesis and angiogenic factors such as vascular endothelial growth factor A and fibroblast growth factor 2 are involved in this process [28]. Endothelial cells proliferate in the developing CL and a fine network of capillary vessels is established in the CL at the mid-luteal stages accompanied by an increased serum progesterone level [28–30]. During luteal regression, apoptosis of luteal cells as well as endothelial cells occur and the number of capillary vessels decreases [31, 32], suggesting a dynamic vascular remodeling is involved in luteolytic process. We confirmed an existence of apoptotic cells in some CL at the late-luteal and early regressed stage, and certain numbers of apoptotic cells were endothelial cells (data not shown). However, capillaries were more visible when pericytes were immunostained for SMA in the CL at the late-luteal stage. We still do not know the role of SMA-positive pericytes in the regulation of luteolysis. On the other hand, some researchers noted an appearance of non-capillary vessels with thickened wall in the regressing CL of various animals including cows [15, 23, 33], that show similar morphology to AVA-like vessels observed in this study.

The appearance of AVA-like vessels in the regressed bovine CL was previously reported by Höfliger [16]; in stages more than 25 days after ovulation, the CL consisted of crooked arteries with the characteristics of AVA. Bauer et al. [33] also noted an increased number of non-capillary vessels with thickened vessel walls in the bovine CL. Hirschberg et al. [34] more recently reported the existence of AVA-like arteries with thickened medial layers showing SMA immunoreactivity during the later luteal stage in cows; however, they reported that “the source of this abundance of vascular smooth muscle-like cells is presently not clear”. We revealed here that SMA-positive fibroblastic cells increased in number in the CL during the late-luteal stage, and accumulated around the arteries distributed in the periphery of the CL and intraluteal connective tissue. It is important to note that a certain population of SMA-immunoreactive cells was positive for vimentin in the CL during the late-luteal stage and proliferation marker, Ki-67, was not associated with an increase of SMA immunoreaction, suggesting that they are resident fibroblasts lying between luteal steroidogenic cells. As luteolysis progressed, the immunoreaction for SMA was restricted to the vascular epithelioid cells with a strong immunoreaction for desmin, a smooth muscle cell-specific intermediate filament. The arterial walls at this stage became thicker and the lumen became narrower, indicating a contractive condition. It is reasonable to assume that the origin of SMA-positive epithelioid cells in AVA-like vessels is myofibroblasts transformed from resident fibroblasts in the CL parenchyma. The AVA-like vessels appearing in association with luteolysis may assist luteolysis by decreasing luteal blood flow in cows.

Ovarian blood flow is known to decrease during luteolysis [4, 12]. Miyamoto et al. [3] clearly demonstrated this change in blood flow during spontaneous and PGF-induced luteolysis in cows. According to their findings, the concentrations of plasma PGF metabolites, indicating the production of uterine PGF, significantly increased 17 days after ovulation, and luteal blood flow concomitantly increased. They also showed that an intramuscular injection of PGF significantly decreased luteal blood flow after 8 h, although a temporal increase of luteal blood flow was observed after 1 h. In the spontaneously regressing CL, luteal blood flow decreases two days after a peak in plasma PGF metabolite concentrations [3]. The accumulation of SMA-positive myofibroblasts around the arteries in the CL during the late-luteal stage and following the establishment of AVA-like vessels in the regressed CL may be involved in decreasing blood flow during spontaneous luteolysis in cows. However, we failed to detect AVA-like vessels in the present PGF-induced regressing CL collected 2–24 h later. We consider that the AVA-like vessels are gradually established in the CL over the repeated estrous cycle because the CL with AVA-like vessels we classified into regressed CL contain the old regressed CL formed more than two cycle before and remained in the ovary. The formation of AVA-like vessels may be observed in the PGF-induced regressing CL collected more than 48 h after a PGF injection.

PGF is a major luteolysin in cows, but does not appear to directly regulate the transformation of fibroblasts to myofibroblasts because the present study failed to show the accumulation of SMA-positive cells or the up-regulation of *Acta2* mRNA expression with PGF-induced luteolysis. TGFβ1 is known to affect the transformation of fibroblasts into myofibroblasts. The results of the present study revealed that the administration of a luteolytic dose of PGF significantly enhanced the expression of *Tgfb1* in the bovine CL. This is consistent with a previous study showing that PGF up-regulated the expression of TGFβ1 [35]. TGFβ1 has been suggested to activate fibroblasts in order to enhance the expression of contractile proteins such as SMA, vimentin, and desmin. Maroni and Davis [36] demonstrated that the treatment of luteal fibroblasts with TGFβ1 induced the expression of laminin, collagen type 1, and matrix metalloproteinase 1, suggesting that TGFβ1 stimulated by PGF is involved in fibrotic changes in the CL during luteolysis. TGFβ1 may influence steroidogenic actions in the bovine CL similar to other TGFβ superfamily members such as activin A and bone morphogenetic proteins [37–39]. Nevertheless, TGFβ1 appears to contribute to the transformation of myofibroblasts during luteolysis in cows in order to establish AVA-like vessels.

## Conclusions

In conclusion, SMA-positive myofibroblasts accumulate around arteries to form AVA-like vessels in the regressing CL of cows. This morphological change in the vasculature appears to be regulated by PGF-induced TGFβ1, and may be involved in vascular occlusion and a reduction in blood flow during luteolysis in cows.

## Abbreviations

AVA: Arteriovenous anastomosis
CL: Corpus luteum
PGF: Prostaglandin F_2α_
qPCR: Quantitative PCR
SMA: α-smooth muscle actin
TEM: Transmission electron microscope
TGFβ1: Transforming growth factor β1
